# Pathway-Based Factor Analysis of Gene Expression Data Produces Highly Heritable Phenotypes That Associate with Age

**DOI:** 10.1534/g3.114.011411

**Published:** 2015-03-09

**Authors:** Andrew Anand Brown, Zhihao Ding, Ana Viñuela, Dan Glass, Leopold Parts, Tim Spector, John Winn, Richard Durbin

**Affiliations:** * Wellcome Trust Sanger Institute, Hinxton, Cambridge, CB10 1SA, United Kingdom; † NORMENT, KG Jebsen Centre for Psychosis Research, Division of Mental Health and Addiction, Oslo University Hospital, Oslo, Norway; ‡ Department of Twin Research and Genetic Epidemiology, King’s College London, St. Thomas’ Campus, Westminster Bridge Road, London SE1 7EH, United Kingdom; § Microsoft Research, Cambridge CB1 2FB, United Kingdom

**Keywords:** aging, factor analysis, gene expression, heritability, linear mixed models

## Abstract

Statistical factor analysis methods have previously been used to remove noise components from high-dimensional data prior to genetic association mapping and, in a guided fashion, to summarize biologically relevant sources of variation. Here, we show how the derived factors summarizing pathway expression can be used to analyze the relationships between expression, heritability, and aging. We used skin gene expression data from 647 twins from the MuTHER Consortium and applied factor analysis to concisely summarize patterns of gene expression to remove broad confounding influences and to produce concise pathway-level phenotypes. We derived 930 “pathway phenotypes” that summarized patterns of variation across 186 KEGG pathways (five phenotypes per pathway). We identified 69 significant associations of age with phenotype from 57 distinct KEGG pathways at a stringent Bonferroni threshold ($P<5.38\times 10^{-5}$). These phenotypes are more heritable ($h^{2}=0.32$) than gene expression levels. On average, expression levels of 16% of genes within these pathways are associated with age. Several significant pathways relate to metabolizing sugars and fatty acids; others relate to insulin signaling. We have demonstrated that factor analysis methods combined with biological knowledge can produce more reliable phenotypes with less stochastic noise than the individual gene expression levels, which increases our power to discover biologically relevant associations. These phenotypes could also be applied to discover associations with other environmental factors.

Aging is a multifactorial process reflecting how the physical state of an organism accumulates changes. Among these, we observe changes in gene expression. Microarrays and more recent RNA-seq technologies allow the simultaneous quantification of cell population average mRNA abundance for thousands of genes. In the case of aging, consistent patterns of age-related changes in gene expression have been observed across several tissues and species (Lu *et al.* 2004), such as overexpression of inflammation and immune-response genes and underexpression of genes involved in energy metabolism in older samples (de Magalhaes *et al.* 2009). Given this commonality of function among genes that show age-related changes in expression, we decided to investigate aging-dependent gene expression in the context of biological knowledge of the function of genes, as provided by pathway annotations.

Array expression experiments generate high-dimensional structured data sets in which there are correlated patterns across large numbers of genes. Some of these are due to known technical or biological effects such as batch effects and cell growth stage, which, when not the focus of the analysis, can be removed by fitting them as covariates. However, even after this, there is typically substantial structural correlation. In previous studies, these can be represented by linear components of expression measurements, or factors, that can be inferred using methods such as principal components analysis (PCA) or factor analysis (Leek and Storey 2007; Parts *et al.* 2011). When the aim is to discover local effects, such as *cis* genetic regulation, the resulting factors can be treated as nuisance variables and removed from further analysis. This has been seen to increase power in analysis (Pickrell *et al.* 2010). Conversely, if the aim is to differentiate between a case and control condition using expression, then factors viewed as global phenotypes could be more effective classifiers than local phenotypes (Hastie *et al.* 2000).

Recently, we applied factor analysis methods in a two-stage procedure to generate phenotypes representing expressions of groups of genes (Stegle *et al.* 2012). After regressing out global factors, as in Parts *et al.* (2011), expression levels for groups of functionally related genes, as defined by annotations from pathway databases, were treated as new expression datasets and the same factor analysis methods were used to construct pathway factors. The factors constructed on pathway sets of genes were taken as concise summaries of common expression variation across each pathway. We tested these factor values as phenotypes and refer to them as phenotype factors or, in some cases, just phenotypes.

Here, we apply this method to gene expression data from abdominal skin tissues from 647 samples. Unlike previous studies that have concentrated on genetic variants that regulate multiple genes within a pathway (Stegle *et al.* 2012), we focus here on discovering associations between gene expression and age. We obtain our pathway gene sets from the Kyoto Encyclopedia of Genes and Genomes (KEGG) pathways (Kanehisa *et al.* 2004). Subsequently, by looking for associations between these new pathway phenotypes and age, we discover groups of functionally related genes with a common response to aging that can be used as biomarkers describing molecular changes with age.

With data from a twin cohort containing both monozygotic and dizygotic twins, we can estimate proportions of variance explained by age, genetic variation, common environmental variation, and unique environmental variation (noise). Stochasticity in gene expression, which will form part of the unique environment component, is believed to play a role in the aging process (Bahar *et al.* 2006). By investigating sources of variation within the pathway phenotypes, we find that they are more robust than the expression of individual genes, with less unique environment variation. This explains some of our success at discovering associations with age.

## Materials and Methods

### Expression profiling

The data analyzed here are part of the MuTHER project (Multiple Tissue Human Expression Resource, http://www.muther.ac.uk/; Nica *et al.* 2011) and were downloaded from the ArrayExpress archive, accession no. E-TABM-1140. In summary, the study included 856 Caucasian female individuals [336 monozygotic (MZ) and 520 dizygotic (DZ) twins] recruited from the TwinsUK Adult twin registry (Moayyeri *et al.* 2012). The age at sampling ranged from 39 to 85 years, with a mean age of 59 years. Punch biopsy samples (8 mm) were taken from relatively photo-protected infra-umbilical skin. Subcutaneous adipose tissue was dissected from each biopsy sample and the remaining skin tissue was weighed and stored in liquid nitrogen. Expression profiling of this skin tissue was performed using Illumina Human HT-12 V3 BeadChips, with 200 ng of total RNA processed according to the protocol supplied by Illumina. All samples were randomized prior to array hybridization and the technical replicates were always hybridized on different BeadChips. Raw data were imported to the Illumina Beadstudio software and probes with fewer than three beads present were excluded. Log2-transformed expression signals were then normalized separately per tissue with quantile normalization of the replicates of each individual followed by quantile normalization across all individuals as previously described (Grundberg *et al.* 2012). Post-QC expression profiles were subsequently obtained for 647 individuals. The Illumina probe annotations were cross-checked by mapping the probe sequence to the NCBI Build 36 genome with MAQ (Li *et al.* 2008). Only uniquely mapping probes with no mismatches and either an Ensembl or a RefSeq ID were kept for analysis. Probes mapping to genes of uncertain function (LOC symbols) and those encompassing a common SNP (1000G, release June 2010) were further excluded, leaving 23,555 probes used in the analysis.

### Gene expression pathway factors

In a two-step approach, factor analysis methods were first used to discover patterns of common variation across the entire dataset. The software package PEER (Parts *et al.* 2011) was applied using the default settings and using technical measurements (experimental batch, RNA quality and concentration) as covariates to create five global factors, which in total explained 35.7% of the variation in the dataset. For each individual, a factor is a weighted sum of all the gene expression measurements of that individual. The weights are chosen so that the factors iteratively explain the maximum amount of variation in the dataset subject to certain prior assumptions; these factors produce concise summaries of consistent patterns of expression for large numbers of genes.

We then used KEGG pathway annotation (186 pathways) as prior information to group genes into pathways. This allows inference of PEER factors for each pathway that we refer to as phenotype factors, in contrast to the global factors previously described. As before, these factors are weighted sums of gene expression measurements, but in this case only of genes within the pathway. Because global factors have been removed from the dataset prior to calculation of phenotype factors, these factors are unlikely to capture global effects on gene expression, but instead capture pathway specific patterns of expression. If a large enough module of genes within the pathway is co-expressed, then one factor will capture the same pattern of co-expression across individuals. Equally, groups of genes could show opposite patterns of expression; this antagonistic gene expression can also be reflected as a factor value that correlates across individuals with one set of genes and is anti-correlated with the other set of genes. Individual genes can contribute positively or negatively to the weighted sum (indicated by the sign of the corresponding weight), meaning that a positive correlation between age and phenotype factor can be induced by negative correlations with individual genes.

We grouped the expression data set into 186 pathway subsets. For each pathway we created five pathway phenotypes using PEER with the default settings. We consider the learned pathway factor values across individuals as five new phenotypes that can be investigated for associations with age. An alternative strategy would be to choose different numbers of factors based on the cumulative amount of variance explained. For the sake of simplicity and as a proof of principle, in this analysis we chose to use five factors because they explained a substantial amount of the variance in expression (17.5%) without too large of a multiple testing burden. The sixth factor, on average, would have explained 2.2% more of the variance.

### Pathway factor and phenotype association

Association tests were performed using the linear mixed models defined in Box 1: between each pathway factor and chronological age and between single genes and chronological age. These models have been implemented by the lme4 package (Bates *et al.* 2014) in R (R Core Team 2013). For each phenotype a likelihood ratio test of the full model, which includes the age term, and the null model (without modeling age) were used to assess evidence for an age effect. *P* values produced by this analysis were assessed for significance, allowing for multiple testing using a Bonferroni-adjusted threshold. Permuted datasets were created that maintained the twin structure by permuting singletons, DZ twins, and MZ twins separately and ensuring that twin pairs were kept together.

Significant associations between phenotype factors and age were further investigated to trace the particular genes within the pathway driving the signal. We report genes with a Bonferroni significant *P* value that accounts for the number of genes within the pathway that was tested.

### Heritability analysis

To compute heritability, the proportion of environmental variance explained by age, and the proportion of variance explained by unique environment, we fitted the full model from Box 1. Then, the genetic component to variation was estimated as twice the additional correlation of MZ twins relative to DZ twins. The environmental component to the phenotype was the sum of the contribution from the fixed age effect, the random noise term, and the shared environmental component, again estimated from the difference between MZ and DZ. Estimates of these proportions are constrained to lie between 0 and 1 inclusive.

**Figure:**
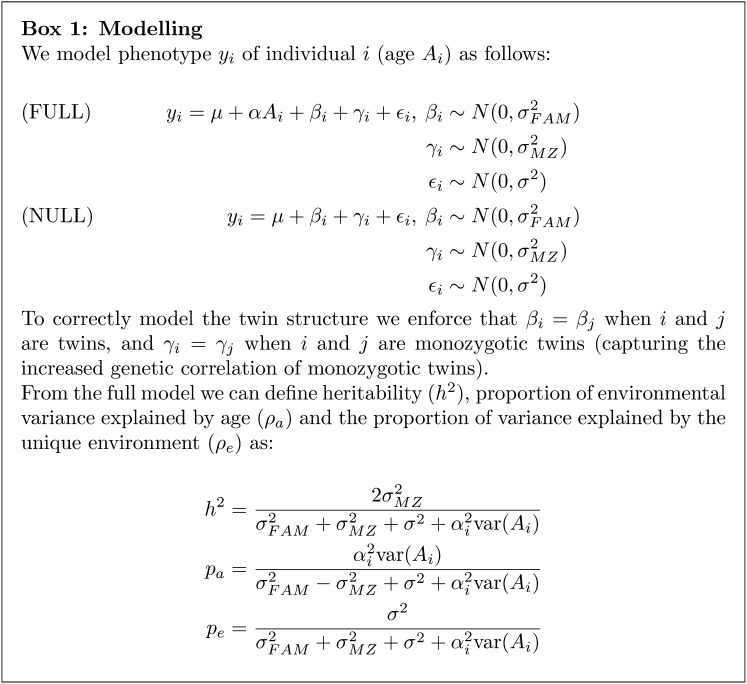


### Single-gene based pathway enrichment analysis

We compared the significant pathways found by our factor analysis methods to those found by looking for enrichment of single gene associations with age. First, we tested each gene for association with age using the methods described in Box 1 and produced a list of Bonferroni significant genes *P*<0.05 (this list contained 682 differentially expressed genes). For each pathway, we applied a Fisher’s exact test to infer whether the proportion of significantly associated genes within the pathway was greater than would be expected by chance. We also investigated whether using an FDR cut-off for significant age associations would produce more significant pathways or whether power would be diluted by including too many false positives. When re-running the analysis using a less stringent threshold (3487 genes were associated with age with FDR<0.05), we found fewer significant pathways and results correlated less well with the results of the factor based analysis [Spearman correlation of 0.36 (*P* = $5.1\times 10^{-7}$) compared to 0.49 for Bonferroni, *P* = $2.1\times 10^{-12}$]. A complete list of all significant single-gene age associations (FDR<0.05; 3487 genes), with estimates of effect size and direction, can be found in Supporting Information, File S1.

## Results

The first stage of the analysis was to remove the effect of both known and unknown nuisance variables from the gene expression data. Using PEER software, we estimated five global factors that explained 35.7% of the variation in the complete gene expression data. Because the aim of this analysis was to find pathway specific responses to aging, we treated these global factors as nuisance covariates and regressed these out of the data, together with batch and RNA quality that are known experimental confounders. Data were then divided into subsets of genes within 186 KEGG pathways that contained more than 10 genes with probes in our dataset. For each pathway, five factors were estimated using PEER as described above, which explained, on average, 17.5% of the residual variation of all genes within this pathway after removing the global factors. For the 186 KEGG pathways, this produced 930 phenotypes that were tested for association with age (see *Materials and Methods* for details). In total, 69 significant associations ($P<5.38\times 10^{-5}$, the Bonferroni-adjusted threshold) from 57 distinct pathways were identified. The most significant 20 pathways are listed in Table 1, and a list of all 57 significant pathways can be found in Table S1.

**Table 1:** List of 20 pathways most significantly associated with age

KEGG_ID	Pathway	*P* of Pathway Factor	No. of Genes in Pathway	Number of Age-Associated Genes	Heritability
00900	Terpenoid Backbone Biosynthesis	6.23$\times 10^{-13}$	13	6	0.00
00980	Metabolism of Xenobiotics by Cytochrome P450	6.47$\times 10^{-13}$	54	6	0.09
01040	Biosynthesis of Unsaturated Fatty Acids	1.11$\times 10^{-12}$	17	6	0.25
00100	Steroid Biosynthesis	1.33$\times 10^{-12}$	14	12	0.41
00650	Butanoate Metabolism	1.51$\times 10^{-12}$	27	8	0.39
04146	Peroxisome	1.56$\times 10^{-12}$	64	17	0.45
00830	Retinol Metabolism	1.93$\times 10^{-12}$	48	6	0.45
00010	Glycolysis Gluconeogenesis	3.59$\times 10^{-12}$	49	12	0.42
00051	Fructose and Mannose Metabolism	3.99$\times 10^{-12}$	32	8	0.32
00290	Valine Leucine and Isoleucine Biosynthesis	1.15$\times 10^{-11}$	11	3	0.00
00561	Glycerolipid Metabolism	2.63$\times 10^{-11}$	38	6	0.34
00620	Pyruvate Metabolism	4.20$\times 10^{-11}$	35	11	0.37
00770	Pantothenate and COA Biosynthesis	4.76$\times 10^{-11}$	16	4	0.48
00280	Valine Leucine and Isoleucine Degradation	5.79$\times 10^{-11}$	35	10	0.51
00020	Citrate Cycle TCA Cycle	1.12$\times 10^{-10}$	23	8	0.43
04916	Melanogenesis	3.34$\times 10^{-10}$	93	10	0.00
04910	Insulin Signaling Pathway	3.70$\times 10^{-10}$	122	13	0.45
00565	Ether Lipid Metabolism	5.89$\times 10^{-10}$	27	3	0.00
00350	Tyrosine Metabolism	9.44$\times 10^{-10}$	32	4	0.34
00640	Propanoate Metabolism	1.03$\times 10^{-9}$	26	6	0.59

List of 20 pathways most significantly associated with age, together with the total number of genes in the pathway, the number of genes within pathways significantly associated with age (P<0.05, corrected using Bonferroni for the total number of genes in the pathway), and the heritability of the pathway factor.

**Figure 1:**
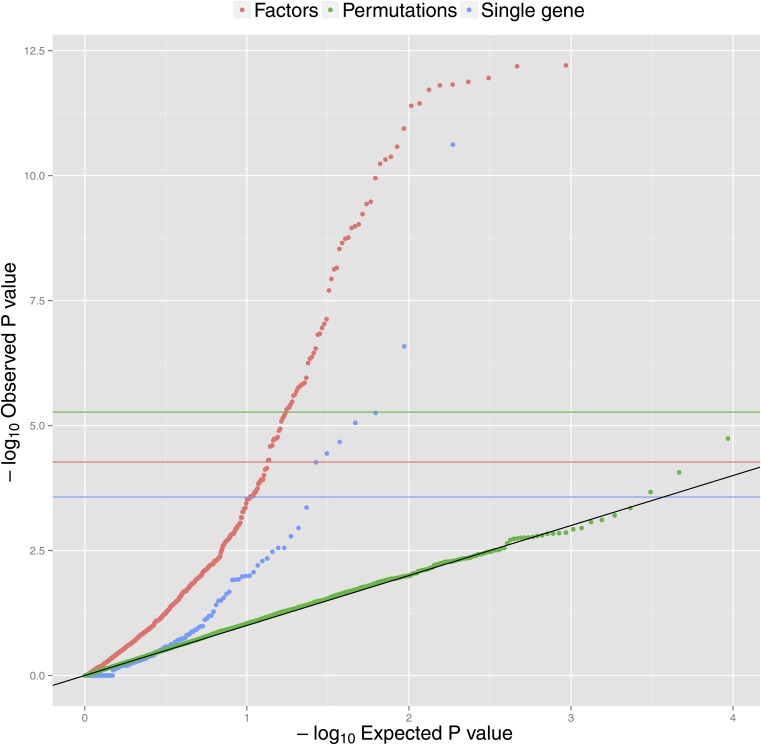
Q-Q plot of observed *P* values against theoretical *P* values for factor analysis (red dots) and single gene–based methods (in blue). Permutations (in green) show the results of a combined analysis of 10 permuted datasets. Horizontal lines show Bonferroni significance thresholds accounting for different numbers of tests (186 tests for single gene measures in blue, 930 for factor analysis in red, and 9300 for the combined 10 permutation analyses in green).

We also explored an alternative method for finding pathway related to aging, looking for enrichment in the number of significantly associated genes falling into a particular pathway, analogous to the method used by the DAVID methodology (Huang *et al.* 2009). This discovered a total of seven significant pathways (Table S2). Thus, applying factor analysis methods to discover significantly associated pathways uncovered eight-times as many hits. All pathways discovered by single gene enrichment methods were also discovered using factor analysis. There is strong concordance between *P* values discovered by the two methods (Spearman correlation = 0.49, *P*$=2.1\times 10^{-12}$). Figure 1 shows a Q-Q plot of *P* values for both methods against the theoretical *P* values under the complete null hypothesis. We see enrichment of significant *P* values for both methods, but this is not present when analyzing the permuted data with factor analysis methods (green dots). This suggests that age plays a widespread role in the expression of these pathways.

To investigate which genes drove the significant pathway associations, we examined how many genes within a significant pathway showed significant age associations (Table 1 and Table S1). On average, 16% of genes within the pathways have P<0.05 after adjusting for the number of genes in the pathway, with a minimum of 1 gene and maximum of 24. The proportion is similar between pathways of different sizes, in contrary to the traditional pathway enrichment analysis, where there is bias toward large pathways.

Different KEGG pathways can contain overlapping sets of genes, because they can describe related biological function. Because of this, our significant associations with age for different pathways could be related as a common underlying effect on a given set of genes. To explore whether the observed age associations are unique to their pathway or common to multiple pathways, we calculated the Spearman correlation between those phenotypes. There are 24 pathway phenotypes with a correlation greater than 0.8 with at least one other phenotype (Table S3). These phenotypes frequently relate to metabolism and form a highly connected set (Figure S1). We infer from this that there could be a common effect of age acting on these phenotype factors. However, these form only a minority of the phenotype factors with significant signal.

**Figure 2:**
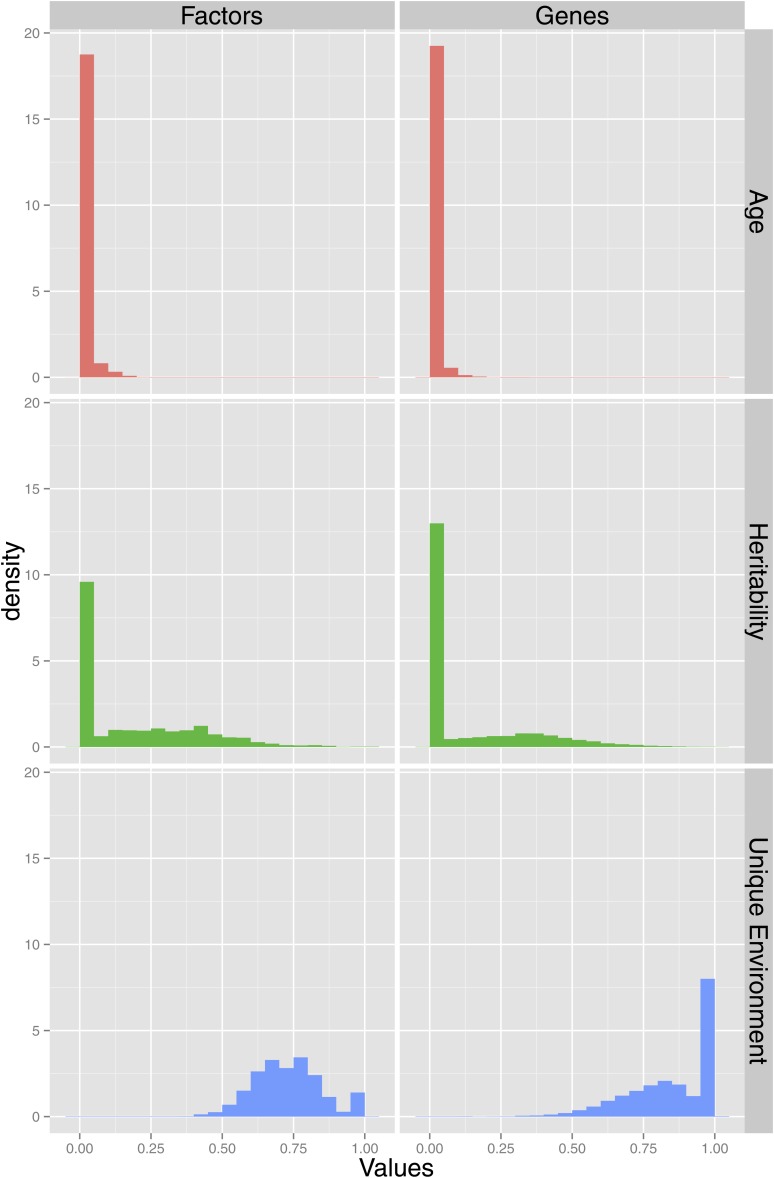
Histograms showing the proportion of environmental variation explained by age, heritability, and the proportion of variance explained by the unique environment for pathway factors and the individual gene measurements.

**Figure 3:**
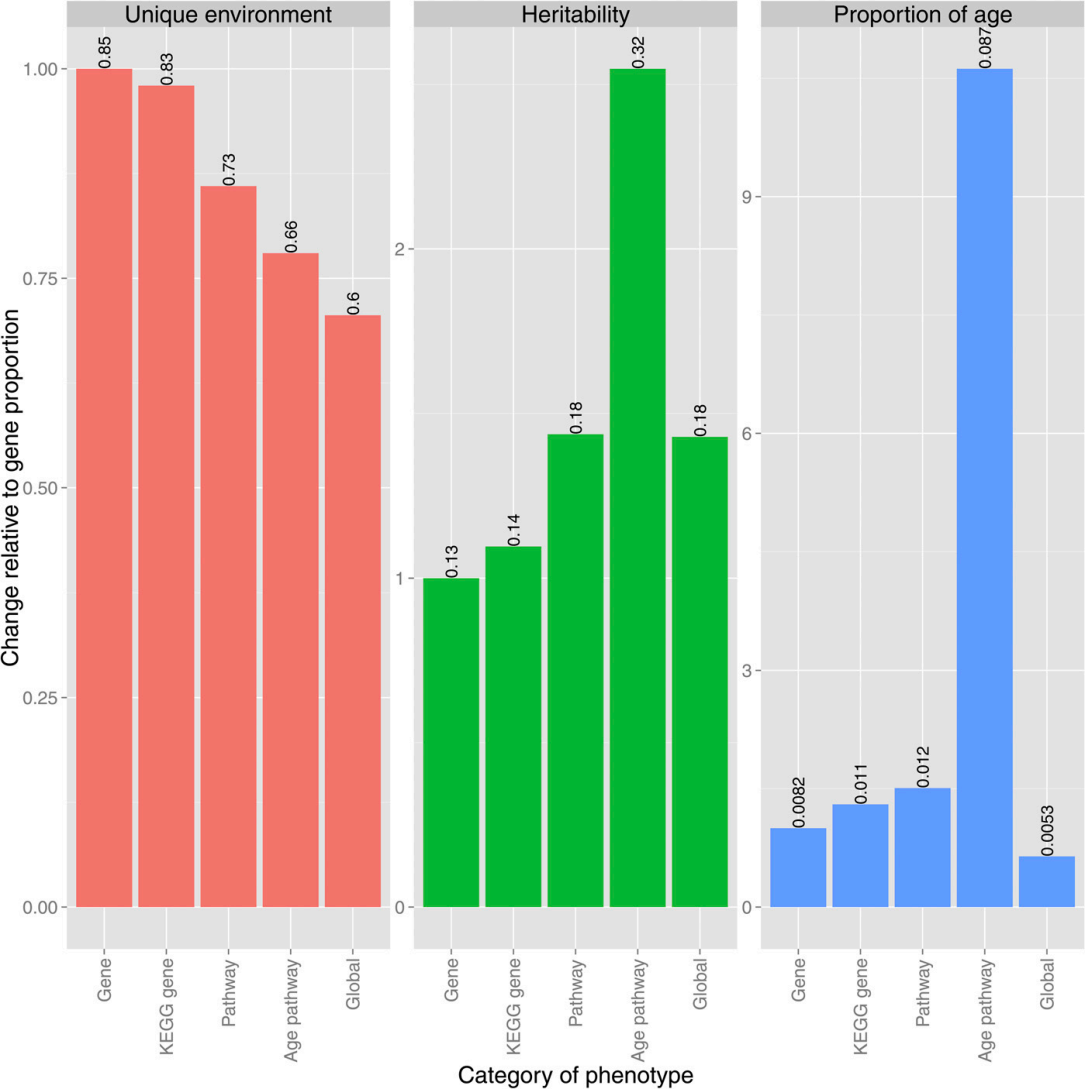
The relative importance of sources of variation to global, pathway, and gene phenotypes. Measures of variation shown are the proportion of variance explained by unique environment, proportion of variance explained by genetics (heritability), and the proportion of environmental variation explained by age. To show more clearly the differences in relative importance of these measures to different classes of phenotypes, all proportions are scaled such that contribution to gene phenotypes equals one. Numbers above the bars give the absolute, unscaled proportions.

We next explored how different sources of variation in the different phenotypes analyzed here affect our ability to discover age associations. We calculated the heritabilities, the proportion of environmental variance explained by age, and the proportion of variance explained by the unique environment (Box 1) for KEGG pathways, global factors (which we have treated as nuisance covariates), and for individual genes (Figure 2, global factor histograms are not shown because there are too few phenotypes). The relative differences in sources of variation between global and pathway factors and the individual genes are shown in Figure 3. We see that as we move away from local phenotypes (individual genes) to pathway phenotypes and then to global phenotypes, the proportion of variation explained by unique environment decreases. This is because there is a stochastic component to each single gene’s expression: by taking a weighted average of a number of genes, we average away this component. If all else were to remain constant, then this reduction in stochastic noise would simultaneously increase heritability (as the total variance decreases) and boost the ability to discover associations with biological meaning, such as age. We see in the first panel of Figure 3 that the relative contribution of unique environment to pathway phenotypes is smaller than the contribution to genes. This also partly explains the results shown in the second and third panels: a greater proportion of variance is explained by age and genetic factors (heritability) for pathway factors than individual gene measurements.

When considering global factors, as expected the unique environment is greatly reduced. However, there is not a strong influence of aging and heritability in this case is still moderate. This is likely because age and genetics do not act in a consistent way across large sets of genes. Leek and Storey (2007) argued that global factors can capture experimental noise and batch effects. This is consistent with our findings. Heritabilities and proportion of variance explained by age for each pathway are reported in Table S4.

We also looked for novel genetic associations with these pathway phenotypes not seen as single gene expression associations. However, this was unsuccessful despite the increased heritability in pathway factors. This is likely due to the genetic architecture of gene regulation. Genes are regulated both in *cis*, where a nearby variant effects the expression of a single gene, and in *trans*, where a long range regulatory effect can hit multiple genes (Grundberg *et al.* 2012). The genetics of pathway phenotypes is a combination of *cis* effects on individual genes and *trans* effects, potentially affecting multiple genes in the pathway. However, *trans* variants typically have much smaller effect size: the increase in the reliability of pathway phenotypes is insufficient to compensate for the lower power to discover *trans* effects. Thus, the only associations discovered were when single genes loaded heavily enough on a pathway to indirectly reflect a *cis* association that could also be detected by a single gene test.

## Discussion

We have seen that both the heritability and the proportion of environmental variance explained by age are greater for pathway phenotypes than for individual genes. Consistent with this, we found a greater proportion of associations for the pathway phenotypes than using single gene tests using this same dataset (Glass *et al.* 2013; 23% compared with 7% of phenotypes are significantly associated with age when using the same 0.05 FDR threshold adopted in that article). This can be explained by our findings regarding the influence of unique environment on pathway phenotypes relative to single genes.

Stochasticity in gene expression, which contributes to the unique environment component that we measure, has been seen to increase with age. For example, animal model studies (Bahar *et al.* 2006; Herndon *et al.* 2002) have reported increased cell-to-cell variation in gene expression with age- and tissue-specific decline of functions associated with stochastic events. Others have found genes associated with longevity to be strongly regulated in older animals with low levels of stochasticity and higher levels of heritability (McCarroll *et al.* 2004; Viñuela *et al.* 2012). The aim of our analysis was to find mean effects rather than variance effects (although both effects are often seen together). By reducing the unique environment variance component using pathway factor analysis methods, we arguably focus much more on systematic longevity changes with age rather than the environmental stochasticity. However, it is difficult to make inference about causality with gene expression: we cannot know whether we are observing changes in expression that are driving the aging process or markers for it. Previous studies have suggested that the latter may be the case, because often changes in gene expression occur in response to aging (de Magalhaes *et al.* 2009).

Of the 57 significant pathways, we frequently see four types of pathway, all of which have been previously linked with aging: insulin signaling; sugar and fatty acid metabolism; xenobiotic metabolism; and cancer-related pathways.

We find the insulin signaling pathway (hsa04910) to be highly associated with age in our data ($P=3.7\times 10^{-10}$). Much evidence has accumulated for the influence of the insulin signaling pathway on longevity, originating in *C. elegans*, where lowered insulin/IGF-1 signaling (IIS) can lead to a significant increase in life span (Friedman and Johnson, 1988). This effect has also been seen in the fruit fly *D. melanogaster* (Clancy *et al.* 2001) and in mice (Holzenberger *et al.* 2003). Outside of model organisms, it has been observed that variants in FOXO transcription factors related to this pathway can affect longevity in humans (Willcox *et al.* 2008).

In addition to those related to insulin, our list of age-associated pathways includes many that are involved in metabolism or glycolosis. Examples of these include biosynthesis of unsaturated fatty acids (hsa00980), butanoate metabolism (hsa00650), glycolysis gluconeogenesis (hsa00010), fructose and mannose metabolism (hsa00051), and valine leucine and isoleucine biosynthesis (hsa00290). It has previously been suggested that metabolism-related pathways play roles in aging and aging-related diseases (Barzilai *et al.* 2012). In particular, Houtkooper *et al.* (2011) showed that glucose and compounds involved in the metabolism of glucose were biomarkers of aging in liver and muscle tissue in mice.

Other aging-related pathways include those involved in the metabolism of xenobiotics that allow cells to deactivate and excrete unexpected compounds. One example is glutathione metabolism (hsa00480, $P=1.45\times 10^{-7}$); glutathione is a well-known antioxidant that protects against cell damage by reactive oxygen species (Pompella *et al.* 2003).

Finally, previous studies have shown that cancer risk is positively associated with age after childhood (Finkel *et al.* 2007; de Magalhães 2013). For example, cellular senescence, when a cell loses the ability to divide, can form a break on cancer development, and clearing such senescent cells can delay the development of age-associated disorders (Baker *et al.* 2011). There are a number of pathways on our list that have been linked to cancer, particularly skin cancer. These include melanogenesis (hsa04916, $P=3.34\times 10^{-10}$), the PPAR signaling pathway (hsa03320, $P=1.83\times 10^{-9}$), the hedgehog signaling pathway (hsa04340, $P=1.12\times 10^{-7}$), and glioma (hsa05214, $P=4.26\times 10^{-7}$)

In addition to age, other phenotypes have been linked to expression patterns of multiple genes. For example, BMI has been linked to expression patterns in adipose tissue of multiple genes within a group that share a common *trans* master regulator, and such phenotypes could mediate between expression and diseases such as type 2 diabetes (Small *et al.* 2011). Principal components and factor analysis have also been suggested as a way to build classifiers for binary traits (Hastie *et al.* 2000), perhaps to predict prognosis of disease from gene expression data. The ability of pathway phenotypes to provide reliable measures of expression with direct biological interpretation means they could also be applied in these situations to understand the relationship between expression and such phenotypes.

Our analysis shows that factor analysis applied to gene expression data effectively reduces stochastic noise in summaries of gene expression patterns, giving more power to discover associations. These phenotypes are substantially more heritable than individual genes. Using them we can improve our ability to identify biological processes underpinning aging. This is consistent with the idea that removing latent factors that exert broad effects on gene expressions increases power in associations. We show that the same idea can be used to create pathway factors that are robust and interpretable. Finally, our analysis reveals pathways that have been seen to be important in longevity from a number of previous studies as well as novel pathways that can be further investigated.
